# Pamoic acid is an inhibitor of HMGB1·CXCL12 elicited chemotaxis and reduces inflammation in murine models of *Pseudomonas aeruginosa* pneumonia

**DOI:** 10.1186/s10020-022-00535-z

**Published:** 2022-09-07

**Authors:** Federica De Leo, Alice Rossi, Francesco De Marchis, Cristina Cigana, Medede Melessike, Giacomo Quilici, Ida De Fino, Malisa Vittoria Mantonico, Chantal Fabris, Alessandra Bragonzi, Marco Emilio Bianchi, Giovanna Musco

**Affiliations:** 1 Biomolecular NMR Laboratory, Division of Genetics and Cell Biology, IRCCS San Raffaele Scientific Institute, Milan, Italy; 2 School of Medicine, Università Vita-Salute San Raffaele, Milan, Italy; 3 Infection and Cystic Fibrosis Unit, Division of Immunology, Transplantation and Infectious Diseases, IRCCS San Raffaele Scientific Institute, Milan, Italy; 4 Chromatin Dynamics Unit, Division of Genetics and Cell Biology, IRCCS San Raffaele Scientific Institute, Milano, Italy

**Keywords:** HMGB1, CXCL12, Inflammation, *Pseudomonas aeruginosa*, Efficacy-testing, Respiratory infection, Mouse model

## Abstract

**Background:**

High-mobility group box 1 protein (HMGB1) is an ubiquitous nuclear protein that once released in the extracellular space acts as a Damage Associated Molecular Pattern and promotes inflammation. HMGB1 is significantly elevated during *Pseudomonas aeruginosa* infections and has a clinical relevance in respiratory diseases such as Cystic Fibrosis (CF). Salicylates are HMGB1 inhibitors. To address pharmacological inhibition of HMGB1 with small molecules, we explored the therapeutic potential of pamoic acid (PAM), a salicylate with limited ability to cross epithelial barriers.

**Methods:**

PAM binding to HMGB1 and CXCL12 was tested by Nuclear Magnetic Resonance Spectroscopy using chemical shift perturbation methods, and inhibition of HMGB1·CXCL12-dependent chemotaxis was investigated by cell migration experiments. Aerosol delivery of PAM, with single or repeated administrations, was tested in murine models of acute and chronic *P. aeruginosa* pulmonary infection in C57Bl/6NCrlBR mice. PAM efficacy was evaluated by read-outs including weight loss, bacterial load and inflammatory response in lung and bronco-alveolar lavage fluid.

**Results:**

Our data and three-dimensional models show that PAM is a direct ligand of both HMGB1 and CXCL12. We also showed that PAM is able to interfere with heterocomplex formation and the related chemotaxis in vitro. Importantly, PAM treatment by aerosol was effective in reducing acute and chronic airway murine inflammation and damage induced by *P. aeruginosa*. The results indicated that PAM reduces leukocyte recruitment in the airways, in particular neutrophils, suggesting an impaired in vivo chemotaxis. This was associated with decreased myeloperoxidase and neutrophil elastase levels. Modestly increased bacterial burdens were recorded with single administration of PAM in acute infection; however, repeated administration in chronic infection did not affect bacterial burdens, indicating that the interference of PAM with the immune system has a limited risk of pulmonary exacerbation.

**Conclusions:**

This work established the efficacy of treating inflammation in chronic respiratory diseases, including bacterial infections, by topical delivery in the lung of PAM, an inhibitor of HMGB1.

**Supplementary Information:**

The online version contains supplementary material available at 10.1186/s10020-022-00535-z.

## Background

High-mobility group box 1 protein (HMGB1) is a highly conserved and ubiquitous nuclear protein (215 amino acids) that acts as a Damage Associated Molecular Pattern promoting inflammation when released in the extracellular space and alarms the immune system. Excessive release of HMGB1 may mediate the systemic inflammatory response syndrome. Immune cells secrete HMGB1 in response to a variety of stimuli, such as pathogen associated molecular patterns (e.g. lipopolysaccharide) and bacterial infections (Lu et al. 2014). Accordingly, high levels of HMGB1 as well as negative correlation between HMGB1 levels and lung function have been described in the context of chronic respiratory diseases, such as chronic obstructive pulmonary disease (COPD) and cystic fibrosis (CF) (Chirico et al. 2015; Gangemi et al. 2015; Liou et al. 2012). Moreover, HMGB1 levels are predictive of time-to-first acute pulmonary exacerbation (APE), number of future APEs within 5 years and time-to-lung transplantation or death in CF patients (Liou et al. 2012). High secretion of HMGB1 has been also observed during *Pseudomonas aeruginosa* infections, such as those associated with COPD and CF, with significant high concentrations in the sputum of CF patients (Liou et al. 2012; Rowe et al. 2008).

Once released by infected epithelial cells, HMGB1 orchestrates responses to tissue damage recruiting neutrophils and impairing bacterial clearance (Bianchi et al. 2017).

Recruitment of inflammatory cells to damaged tissues after infection or injury relies on the interaction of the fully reduced form of HMGB1 with the chemokine C-X-C Motif Chemokine Ligand 12 (CXCL12). The HMGB1·CXCL12 complex in turn promotes C-X-C chemokine receptor type 4 (CXCR4)-dependent recruitment of inflammatory cells to injured tissues (Schiraldi et al. 2012), which exacerbates the immune response in pathological conditions (D’Agostino et al. 2018). Signaling via the CXCL12/HMGB1/CXCR4 axis is pervasive: CXCL12 is expressed by all cells, and for this reason it is deemed a homeostatic chemokine (Mezzapelle et al. 2022; Pawig et al. 2015). HMGB1 as well is expressed by all cells, but will interact with CXCL12 only when it is actively released in the microenvironment by severely stressed cells (Bianchi et al. 2017); finally, CXCR4 is expressed by most cells, albeit at different levels (Mezzapelle et al. 2022; Pawig et al. 2015).

Notably, during *P. aeruginosa* infection neutralization of HMGB1 in the lung with monoclonal antibodies resulted in significant protection, with reduction of neutrophil recruitment and lung injury in both *cystic fibrosis transmembrane conductance regulator* deficient (*Cftr*^−/−^) and wild-type mice (Entezari et al. 2012). However, effective, systemic intraperitoneal (i.p.) delivery of monoclonal antibodies would be expensive and impractical for chronic treatments. Hence, the pharmacological targeting of HMGB1 with small molecules could be a valuable alternative in the treatment of inflammation in chronic respiratory diseases and in pulmonary bacterial infections. In the past, we have shown that HMGB1 is druggable with small molecules such as glycyrrhizin (Mollica et al. 2007) and salicylates: salicylic acid (Choi et al. 2015), Diflunisal (De Leo et al. 2019), 5,5′-methylenedi-2,3-cresotic acid (MCA) (De Leo et al. 2020). All these molecules inhibit HMGB1 chemoattractant activity through the disruption of its heterocomplex with CXCL12 (HMGB1·CXCL12) and impairment of the HMGB1/CXCL12/CXCR4 axis.

We then asked whether other salicylates could effectively inhibit the detrimental HMGB1 activity in bacterial pulmonary infections. We focused our attention on pamoic acid (PAM), a solubilizer commonly used in drug formulations and which is not absorbed across mucosae and by the oral route (Neubig 2010; Zhao et al. 2010). We show here that PAM directly binds to both HMGB1 and CXCL12, disrupts their heterocomplex, herewith inhibiting HMGB1·CXCL12 dependent chemotaxis in vitro. Importantly, PAM delivered by aerosol in vivo to C57BL/6NCrlBR mice with *P. aeruginosa* infection shows no toxicity in the airways and can ameliorate neutrophilic inflammation and lung damage.

These results show for the first time a promising efficacy of a small molecule inhibitor of HMGB1-dependent inflammation in mouse models of acute and chronic *P. aeruginosa* respiratory infection.

## Materials and methods

### Ethics statement

Animal studies adhered strictly to the Italian Ministry of Health guidelines for the use and care of experimental animals (protocol #733). Research with the *P. aeruginosa* multidrug-resistant (MDR)-RP73 isolate from a CF individual and storage of biological materials were approved by the Ethics Commission of Hannover Medical School, Germany.

### Proteins expression and purification

Recombinant HMGB1 constructs (Accession code P63158, residues 1–215 and BoxA, residues 1–89) and recombinant human CXCL12 in labeled and unlabeled forms were produced as described (De Leo et al. 2019). Proteins used for cell-based assays were provided by HMGBiotech (Milan). Pamoic acid was purchased from Sigma Aldrich.

### Nuclear Magnetic Resonance (NMR) experiments

After expression and purification, HMGB1 was dialyzed against NMR buffer, containing 20 mM phosphate buffer pH 7.3, 150 mM NaCl, and 1 mM DTT. CXCL12 was dialyzed against a buffer containing 20 mM phosphate buffer pH 6, 20 mM NaCl. Protein concentrations were determined considering molar extinction coefficients at 280 nm of 21,430 and 8700 M^−1^ cm^−1^ for HMGB1 and CXCL12, respectively. NMR spectra were recorded at 298K on a Bruker Avance 600 MHz spectrometer (Karlsruhe, Germany) equipped with a triple-resonance TCI cryoprobe with an x, y, z-shielded pulsed-field gradient coil. Spectra were processed with TopspinTM 3.2 (Bruker) and analyzed with CcpNmr Analysis 2.4 (Vranken et al. 2005). Details on PAM resonance assignments, on Saturation Transfer Difference (STD) and Water-Ligand Observed via Gradient Spectroscopy (WaterLOGSY) experiments, on NMR titrations and on line-shape analysis are reported in Additional file 1.

### Data driven docking models and molecular images

Molecular docking of PAM on CXCL12 (PDB 4UAI), on BoxA (residues G3-Y77) and on BoxB (A93-G173), whose structures were extracted from 2YRQ (first structure of the NMR bundle), were performed using the data-driven docking software HADDOCK 2.2 (Dominguez et al. 2003; Van Zundert et al. 2016) and following the classical three-stage procedure, which includes: (1) randomization of orientations and rigid body minimization, (2) simulated annealing in torsion angle space, and (3) refinement in Cartesian space with explicit water. Details on docking calculations, Ambiguous interaction restraints (AIRs) and cluster analysis are reported in Additional file 1.

Molecular images were generated by 3D Protein Imaging online server (Tomasello et al. 2020). CSP plots were produced with Xmgrace program (http://plasma-gate.weizmann.ac.il/Grace/).

### Cell migration experiments

For fibroblast chemotaxis, modified Boyden chambers were used with filters (pore diameter 8 μm; Neuro Probe) coated with 50 μg/mL fibronectin (Roche). Mouse 3T3 cells (50,000 in 200 μL) were added to the upper chamber. Serum-free DMEM as negative control, HMGB1, and/or PAM were added to the lower chamber at the indicated concentrations, and then cells were left to migrate for 3 h at 37 °C. Cells were fixed with ethanol and stained with Giemsa Stain (Sigma), then non-migrating cells were removed with a cotton swab. All assays were done at least in biological triplicate and were repeated at least twice. The migrated cells were acquired with Zeiss Imager M.2 microscope at 10× magnification, then evaluated with an automated counting program.

### Mouse models of acute and chronic *P. aeruginosa* infection

*Pseudomonas aeruginosa* strains included PAO1 and MDR-RP73 strains isolated from a CF patient’s airways. Immunocompetent C57Bl/6NCrlBR male mice (8–10 weeks, Charles River Calco, Italy) were challenged with 1 × 10^6^ colony-forming units (CFUs) of the planktonic PAO1 strain for acute infection or with 5 × 10^5^ CFUs of the MDR-RP73 strain embedded in agar beads for chronic infection by intratracheal inoculation (i.t.), as previously described (Bragonzi et al. 2009; Cigana et al. 2016; Facchini et al. 2014). Mice were treated with PAM (1 mM and 3 mM) or vehicle (PBS) by local pulmonary administration using the Penn-Century MicroSprayer^®^ Aerosolizer with treatment schedules established previously (Cigana et al. 2020). In the acute infection model, the treatment schedule used with PAM was a single dose administered after infection. In the chronic infection model daily doses repeated for 7 days were administered. Body weight and health status were monitored daily. CFU counts, and cell counts in the bronchoalveolar lavage fluid (BALF) were analyzed as previously described (Cigana et al. 2016; Facchini et al. 2014; Kukavica-Ibrulj et al. 2014) at 6 h after acute infection or 7 days after chronic infection. Myeloperoxidase (MPO) and neutrophil elastase were measured in the BALF and lung homogenates by ELISA (R&D DuoSet ELISA Development System). Additional details in accordance with the Animal Research: Reporting of In Vivo Experiments guidelines (Kilkenny et al. 2010) are reported in Additional file 1.

### Statistics

Statistical analyses were performed with GraphPad Prism using one-way ANOVA plus Dunnett’s post-test for in vitro data, two-way ANOVA with Bonferroni Multiple Comparison test for body weight change and temperature and Kruskal–Wallis test with Dunn’s Multiple Comparison test for the other in vivo readouts.

## Results

### PAM binds HMGB1 and CXCL12 and inhibits the HMGB1·CXCL12 heterocomplex in vitro

**Fig. 1:**
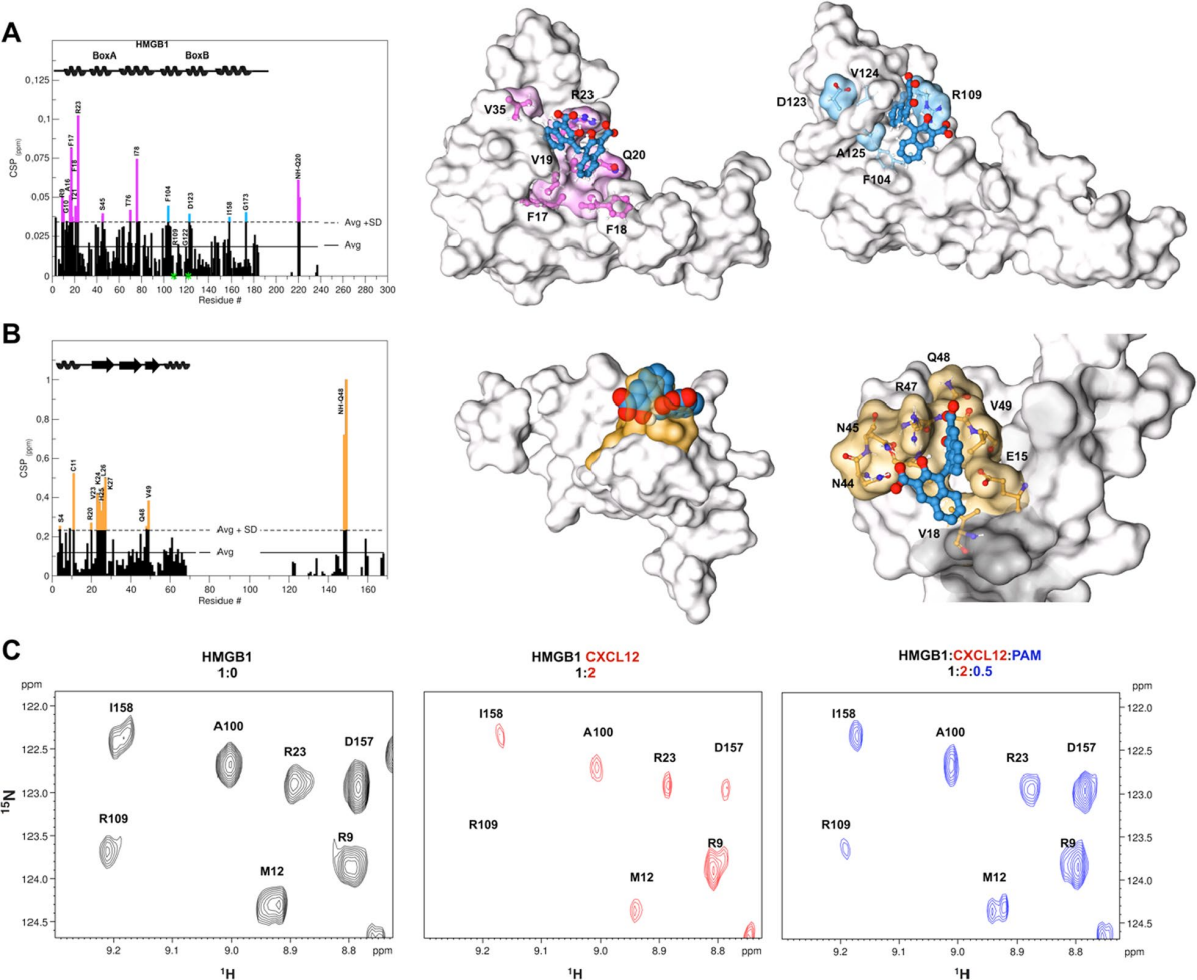
PAM directly binds to HMGB1 and CXCL12 and disrupts their heterocomplex. **A** Histogram showing residue-specific CSPs of ^15^N-HMGB1 (~ 0.1 mM) upon addition of equimolar ratio of PAM (helices are schematically represented on top). Missing residues are prolines or are absent because of exchange with the solvent. BoxA and BoxB residues with CSP > Avg + SD are represented in magenta and light blue, respectively. HADDOCK models of interaction of PAM (licorice representation) with BoxA (middle) and BoxB (right) (gray surface and colored residues with CSP > Avg + SD). HMGB1 residues (sticks) involved in hydrophobic and electrostatic interactions with PAM are labeled. **B** Histogram showing the CSPs of ^15^N-CXCL12 amides (~ 0.1 mM) upon addition of equimolar ratio of PAM. Missing residues are prolines. Elements of secondary structure are represented on top. Middle: HADDOCK model of interaction of PAM (CPK representation) with CXCL12 (gray surface). CXCL12 residues with CSP > Avg + SD located around the sY21 binding site are in orange. Right: Zoom in the binding site, CXCL12 residues (sticks) involved in hydrophobic and electrostatic interactions with PAM (sticks) are explicitly labeled. **C** Selected region of ^1^H-^15^N HSQC HMGB1 (0.1 mM) spectrum without (black, left), with 0.2 mM CXCL12 (red, middle) and upon addition of 0.05 mM PAM (blue, right)

To investigate whether PAM is a direct ligand of HMGB1 we first used a battery of Nuclear Magnetic Resonance (NMR) binding experiments including Saturation Transfer Difference (STD), Water-Ligand Observed via Gradient SpectroscopY (waterLOGSY) and chemical shift perturbation (CSP). STD effects, inversion of the sign in waterLOGSY spectra and overall line broadening effects of PAM (1 mM) in the presence of 50 µM HMGB1 first indicated a protein–ligand interaction (Additional file 1: Figure S1). NMR titrations of ^1^H-^15^N labeled HMGB1 with PAM confirmed binding, with a specific set of amide resonances significantly shifting (CSP > Avg + SD) or disappearing upon addition of increasing concentrations of PAM (up to 0.15 mM) (Additional file 1: Figure S2). Residues whose amides were mostly affected by the binding (R9, G10, A16, F17, Q20, T21, R23, S45, F104, R109, G122, D123) when mapped on BoxA and BoxB structures (Fig. 1A) defined a small pocket on the short arm of the L-shaped fold of both HMG boxes (Fig. 1A). This interaction surface was well in line with the one previously reported for other HMGB1 inhibitors (De Leo et al. 2019). The binding occurred in the fast-intermediate exchange regime on the NMR time scale, in agreement with the apparent K_d_ of 170 ± 17 µM obtained by lineshape analysis of the peaks in ^1^H-^15^N HSQC spectra as a function of added ligand (Waudby et al. 2016). The residues with significant CSPs were then used to generate three-dimensional data-driven docking models of PAM in complex with BoxA and BoxB (Fig. 1A). Both models highlighted a central clamping electrostatic interaction between the PAM carboxylate and the guanidinium groups of R23 and R109 on BoxA and BoxB, respectively. The interaction was further stabilized by van der Waals (vdW) contacts between the aromatic rings of PAM and the hydrophobic sidechains of residues on BoxA (F17, F18, V19, V35) and BoxB (F104, V124, A125) (Fig. 1A, Additional file 1: Figures S3, S4). The models agreed with the ligand epitope mapping via STD buildup experiments and intermolecular Nuclear overhauser effects (NOEs), that indicated proximity between the aromatic protons of PAM (H2, H3 and H4) and HMGB1 (Additional file 1: Figure S5 and Table S1).

We have recently shown that HMGB1 ligands often interact with CXCL12. Indeed, NMR titrations of ^15^N-CXCL12 with PAM indicated binding (apparent K_d_ of 66 ± 4 µM), with significant CSPs affecting residues V23, K24, H25, L26, K27, Q48, V49 (Fig. 1B, Additional file 1: Figure S6). A data-driven docking model suggested that PAM, similarly to diflunisal and MCA (De Leo et al. 2019, 2020), binds in the so-called CXCR4 sulfotyrosine (sulfoY21) binding site (Veldkamp et al. 2008). The PAM carboxylate establishes polar interactions with the amide side chains of N44 and N45, the hydroxyl group creates an H-bond with the guanidinium group of R47, the second carboxylate has polar interactions with Q48 backbone atoms, and the aromatic rings establish stabilizing hydrophobic interactions with V49 and V18 (Fig. 1B, Additional file 1: Figure S7).

Next, by NMR-based Antagonist Induced Dissociation Assay (Krajewski et al. 2007) we proved that PAM disrupts the HMGB1·CXCL12 heterocomplex. We acquired ^15^N HSQC spectra of the free ^15^N-HMGB1 (Fig. 1C black contours) and of a preformed complex of ^15^N-HMGB1 (0.1 mM) with unlabeled CXCL12 (0.2 mM) (Fig. 1C red contours). The latter displayed evident line broadening and reduction of the ^15^N-HMGB1 peaks (Fig. 1C) due to the complex intermediate exchange regime on the NMR time scale (De Leo et al. 2019). As already observed in NMR titrations with other salicylates (De Leo et al. 2019, 2020), low stoichiometric ratios of PAM induced visible line broadening effects on the ^15^N HSQC spectrum of HMGB1 in complex with CXCL12, possibly due to multiple equilibria occurring in solution (Additional file 1: Figure S8). However at 0.5 stochiometric ratio we observed a significant recovery of the majority of the ^1^H-^15^N resonances in the ^1^H-^15^N HSQC spectrum of HMGB1 (Fig. 1C, Additional file 1: Figure S8), indicating that the complex was partially disrupted.

Collectively, these data indicate that PAM is a direct ligand of both HMGB1 and CXCL12 and is able to interfere with heterocomplex formation.

### PAM inhibits HMGB1·CXCL12 dependent chemotaxis

**Fig. 2:**
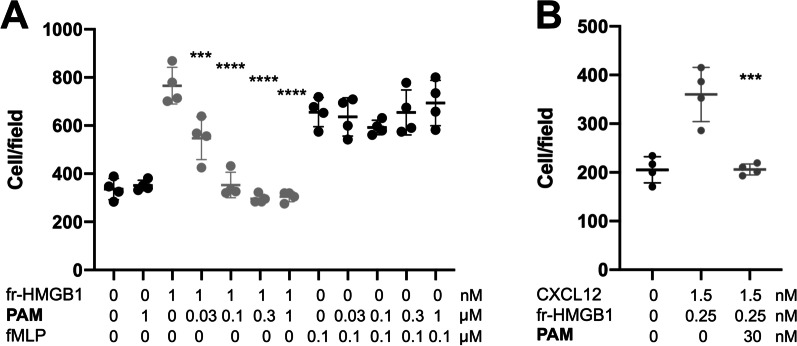
**A** PAM inhibits HMGB1-induced, but not fMLP-induced cell migration. Mouse 3T3 fibroblasts were subjected to chemotaxis assays in Boyden chambers, 1 nM fr- (fully reduced) HMGB1, or no chemoattractant was added in the lower chamber, together with the indicated concentrations of PAM. Data points with average ± standard deviation (Avg ± SD; n = 4, each point represents a biological replicate) in a representative experiment. Statistics: one-way ANOVA (P = 0.0001), followed by Dunnett’s post-tests. ***P < 0.0004, ****P < 0.0001 relative to no PAM addition. PAM does not inhibit chemotaxis toward fMLP. Data points (n = 4) with Avg ± SD in one representative experiment (each point represents a biological replicate). Migration in the absence or in the presence of the indicated concentration of PAM is not statistically significant (Statistics: one-way ANOVA (P = 0.96). **B** PAM inhibits chemotaxis toward the HMGB1**·**CXCL12 heterocomplex. Data points (n = 3) with Avg ± SD in one representative experiment (of three performed in different days). Migration in the presence or absence of PAM is significantly different (P = 0.0002, one-way ANOVA plus Dunnett's post-test; ***P < 0.0004 relative to no PAM addition)

Next, we asked whether PAM was able to inhibit the chemotaxis elicited by HMGB1 and by the HMGB1·CXCL12 heterocomplex. Indeed PAM reduced the HMGB1-dependent migration of 3T3 mouse fibroblasts in a dose-dependent way with an IC_50_ of about 30 nM (Fig. 2A). The inhibition of HMGB1 chemotactic activity was specific, as PAM did not affect chemotaxis toward fMLP (Fig. 2A). As expected, PAM also inhibited chemotaxis induced by HMGB1·CXCL12 (Fig. 2B). Taken together these data indicate that PAM is a very effective inhibitor of the chemotactic activity HMGB1·CXCL12 heterocomplex in vitro.

### PAM reduces inflammation and tissue damage in a murine model of acute *P. aeruginosa* pneumonia

**Fig. 3:**
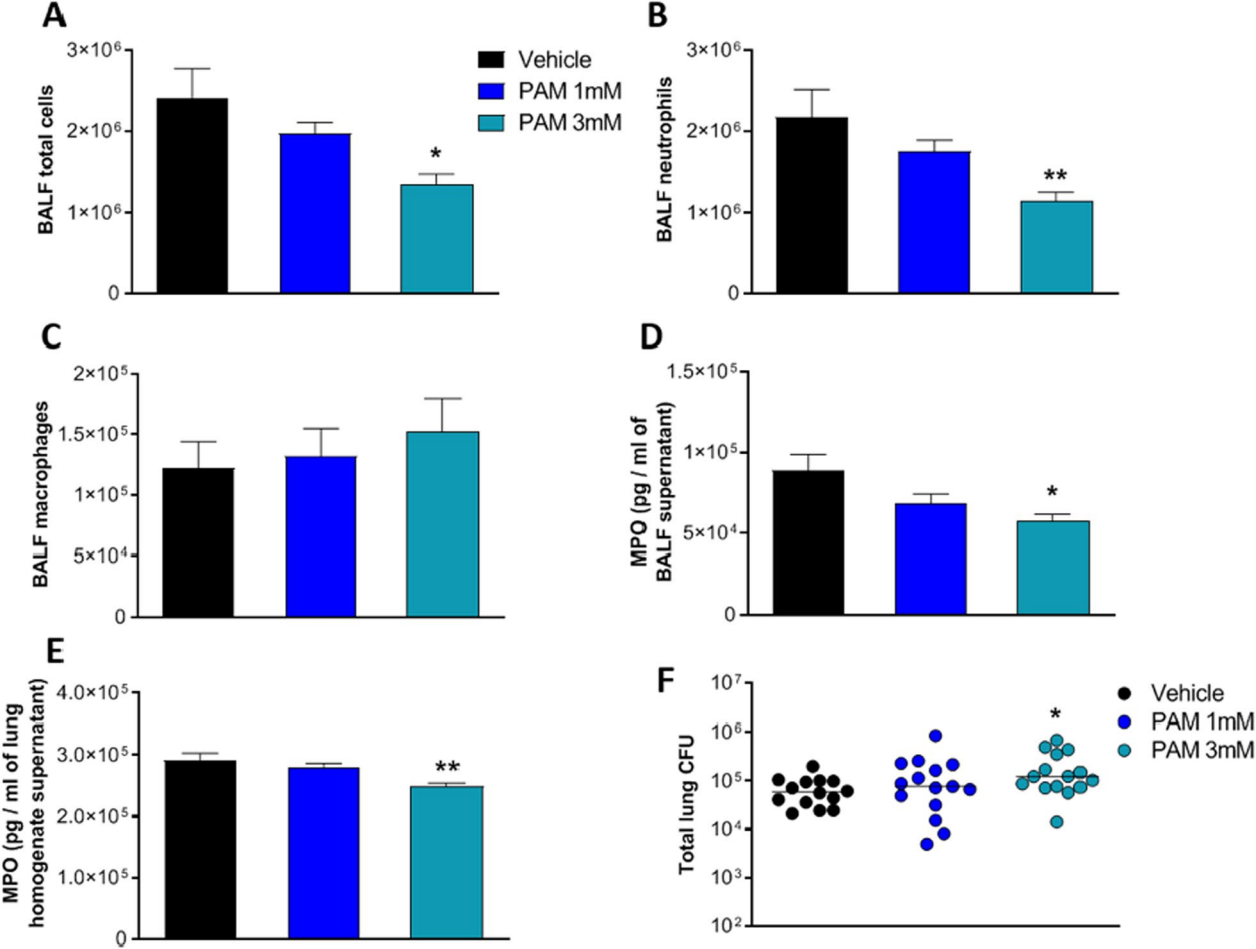
Efficacy of aerosol treatment with PAM (1 mM and 3 mM) in a mouse model of acute *P. aeruginosa* airway infection. C57BL/6NCrlBR male mice (aged 8–10 weeks) received intratracheal inoculation with 1 × 10^6^ CFUs of planktonic PAO1 strain. Five minutes after infection, PAM (1 mM, or 3 mM or vehicle) were administered via an aerosolizer. After 6 h, mice were sacrificed, bronchoalveolar lavage fluid (BALF) was collected, and the lungs were excised and homogenized. Total cells (**A**), neutrophils (**B**) and macrophages (**C**) were counted on BALF. BALF and lung homogenates were centrifuged. MPO concentration was evaluated in the supernatants of BALF (**D**) and lung homogenate (**E**) by ELISA assay. Data are presented as mean ± SEM. BALF and lung homogenates were plated on tryptic soy agar to determine the bacterial burden (**F**). Each dot represents total CFUs per lung from one mouse, and horizontal lines represent the median values. Data are pooled from three independent experiments (n = 14–15 mice). Statistical significance is indicated: *P < 0.05; **P < 0.01

Since PAM inhibits in vitro the HMGB1·CXCL12 elicited chemotaxis, we asked whether it could impair neutrophil recruitment in a murine model of acute *P. aeruginosa* respiratory infection (Bragonzi 2010; Cigana et al. 2020). First, we tested toxicity in C57BL/6NCrlBR mice with two different doses (1 and 3 mM) of PAM by aerosol in repeated daily administrations for 7 days. Several readouts including weight loss, body temperature and health status after treatment did not show significant changes between PAM and vehicle (Additional file 1: Figure S9 and data not shown). Next, C57BL/6NCrlBR mice were challenged with planktonic *P. aeruginosa* PAO1 strain by i.t. inoculation to induce acute infection. Local treatment via the aerosol route with 1 and 3 mM PAM started 5 min after infection and was compared with vehicle (PBS). To define the effect of PAM on the airway inflammatory response, we measured leukocyte recruitment in the BALF 6 h after infection. A single dose of PAM reduced total cells, particularly neutrophils, in a dose-dependent manner with statistical significance at 3 mM PAM compared to vehicle (Fig. 3A and B). No significant difference was observed in macrophage numbers (Fig. 3C). Next, MPO and neutrophil elastase, markers of neutrophil infiltration and tissue damage (Haegens et al. 2008), were measured by ELISA. MPO levels in BALF and lung were significantly reduced in mice treated with 3 mM PAM compared to vehicle (Fig. 3D and E). Conversely, neutrophil elastase both in BALF and lung supernatant was not affected by PAM treatment (Additional file 1: Figure S10A and B). When bacterial burdens in the lung were evaluated, moderately but significantly higher CFUs were found in mice treated with 3 mM PAM in comparison to those treated with vehicle (Fig. 3F). No difference in the CFUs was observed between 1 mM PAM and vehicle. Our results indicate that a single dose of PAM treatment by aerosol was effective in reducing acute airway murine inflammation and damage induced by *P. aeruginosa* in a dose dependent manner. However, PAM efficacy was associated with a modest increase in bacterial burden, indicating a low risk of acute pulmonary exacerbation in this murine model.

### PAM reduces inflammatory response but controls bacterial burden in a mouse model of chronic *P. aeruginosa* pneumonia

Next, we tested the efficacy of repeated doses of PAM in treating chronic lung infections.

**Fig. 4:**
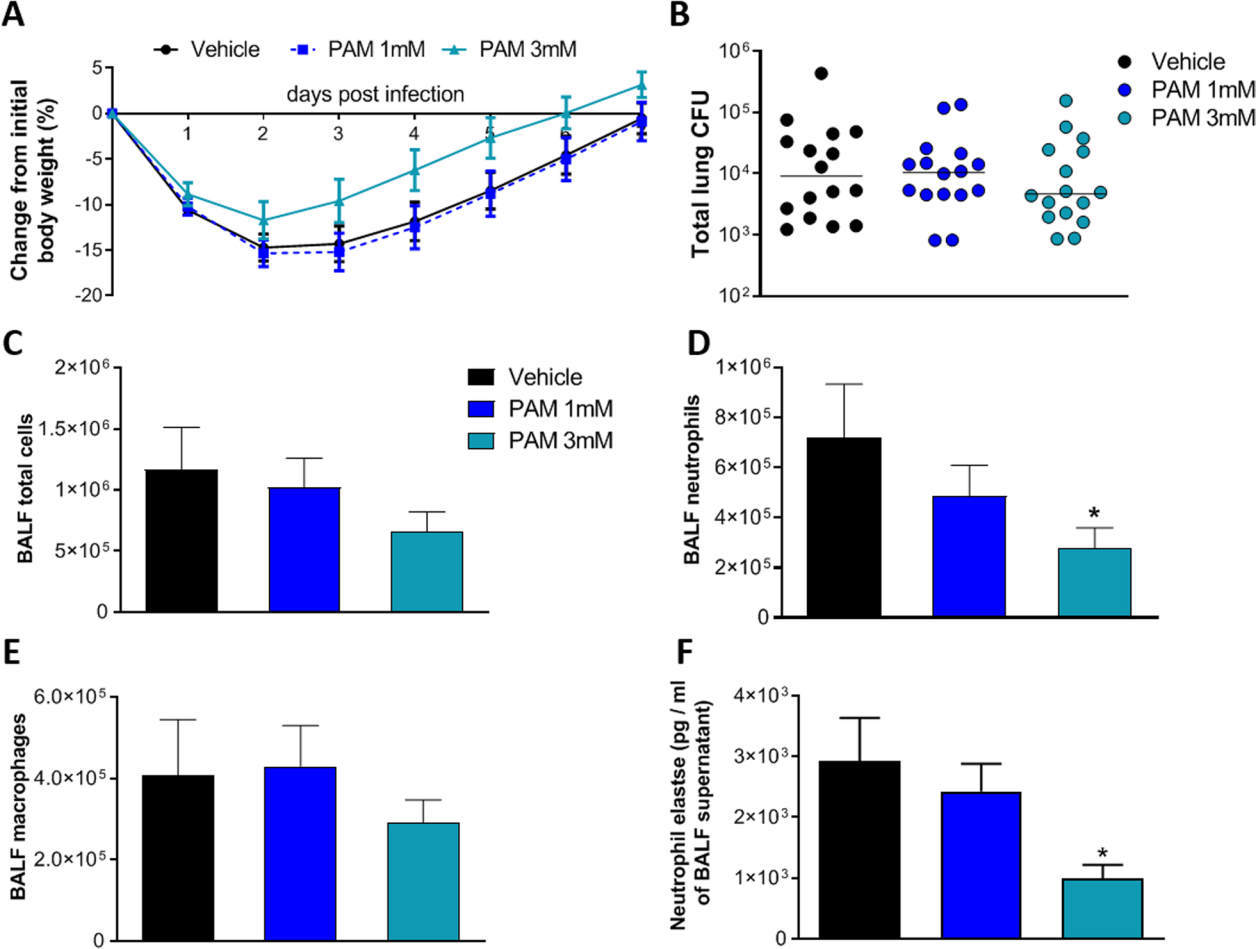
Efficacy of aerosol treatment with PAM (1 mM and 3 mM) in a murine model of *P. aeruginosa* MDR-RP73 chronic airways infection. C57BL/6NCrlBR male mice (aged 8–10 weeks) received intratracheal inoculation with 5 × 10^5^ CFU of MDR-RP73 strain embedded in agar beads. Treatment started 5 min after infection, with PAM (1 mM and 3 mM) or vehicle administered via aerosol by Penn Century daily for 7 days. Before each administration, mice were weighted, and the percentage change from the initial body weight was averaged for each group of mice (**A**). Data are presented as mean ± SEM. At day 7 post-infection, mice were sacrificed, BALF was collected and lungs were excised and homogenized. BALF and lung homogenates were plated on tryptic soy agar to determine the bacterial burden (**B**). Each dot represents total CFU per lung from one mouse, and horizontal lines represent the median values. Total cell (**C**), neutrophil (**D**) and macrophage (**E**) counts were performed on BALF. Neutrophils elastase concentration was evaluated in the supernatants of BALF (**F**) by ELISA assay. Data are presented as mean ± SEM. Data are pooled from three independent experiments (n = 15–16 mice). Statistical significance is indicated: *P < 0.05

To mimic a chronic infection, mice received i.t. inoculations of the *P. aeruginosa* MDR-RP73 strain embedded in agar beads (Facchini et al. 2014). Administration via the aerosol route with 1 mM, 3 mM PAM or vehicle (PBS) was started five minutes after infection and was repeated daily for seven administrations. Over the course of 7 days, mice treated with 3 mM PAM exhibited less loss and faster recovery of body weight than vehicle-treated mice (Fig. 4A). No significant differences in bacterial load were observed after treatment with PAM (1 and 3 mM) or vehicle, indicating that this schedule of treatment does not affect the chronic *P. aeruginosa* infection (Fig. 4B).

Treatment with 3 mM PAM reduced the total cell counts (Fig. 4C) with significant difference in the number of neutrophils in BALF when compared to vehicle, indicating a reduction of inflammation (Fig. 4D). No differences were observed in the number of macrophages (Fig. 4E). Cellular recruitment in mice treated with 1 mM PAM followed the same trend, but differences with vehicle did not reach statistical significance. Next, to validate these results, MPO levels and neutrophils elastase in BALF and lung supernatant were measured. Neutrophils elastase levels in BALF supernatant were significantly reduced in mice treated with 3 mM PAM compared to vehicle (Fig. 4F). No difference in neutrophil elastase were observed in the supernatant of lung homogenate after PAM treatment (Additional file 1: Figure S11C). MPO levels were similar in PAM treated mice compared to vehicle (Additional file 1: Figure S11A and B). Our results indicate that repeated doses of PAM treatment by aerosol were effective in reducing chronic airway murine inflammation induced by *P. aeruginosa* in a dose dependent manner without affecting bacterial burden.

## Discussion

Increasing evidences suggest that inhibition of the alarmin HMGB1 in *P. aeruginosa* infection could offer a potential therapeutic strategy to reduce bacterial infection and lung inflammation. Previous research showed that delivery of monoclonal antibodies (mAB) against HMGB1 conferred significant protection against *P. aeruginosa* infection, neutrophil recruitment and lung injury in mouse models of CF (Entezari et al. 2012). In addition, delivery of recombinant BoxA, an HMGB1 antagonist, is effective in reducing Toll like receptor 4 (TLR4), Receptor for advanced glycation (RAGE), and inflammatory cytokines levels in the cornea of *P. aeruginosa*-infected mice (Ekanayaka et al. 2018).

Importantly, recent work in our groups has also shown that HMGB1 is druggable and that its ability to recruit immune cells upon injury can be modulated by small molecules (De Leo et al. 2019, 2020), a strategy that appears more convenient than inhibition through mAb or biologics. In particular, salicylates appear to be well suited in impairing the HMGB1·CXCL12 heterocomplex, through direct targeting of both HMGB1 and CXCL12; they effectively inhibit chemotaxis via the HMGB1/CXCL12/CXCR4 axis (De Leo et al. 2019, 2020). During a structure–activity relationship study aiming at identifying salicylate derivatives with improved HMGB1 inhibition activity, we were intrigued by the physical–chemical properties of PAM (Nazir et al. 2019). This molecule (also known as Embonic Acid) does not cross lipid membranes and cannot traverse mucosal barriers, and is thus well-suited for aerosol local delivery. Importantly, PAM is already used in drug formulations (Chue and Chue 2012; Song et al. 2016) and might therefore benefit from a fast track for approval by regulatory agencies.

Here we have shown that PAM, similarly to other salicylates, is a direct ligand of both HMGB1 and CXCL12. The pattern of interaction of PAM with the single HMG Boxes and with CXCL12 is highly reminiscent of the one observed for Diflunisal (De Leo et al. 2019) and MCA (De Leo et al. 2020). PAM appears appropriate to interact with both HMG boxes, as it fulfills three out of the four pharmacophoric requirements previously defined for HMGB1 ligands, consisting of two hydrophobic and two H-bonding acceptor features (De Leo et al. 2020). In particular, data driven docking models indicate that major interactions between the ligand and the target consist of a salt bridge between one carboxylate of PAM and the guanidinium groups of the conserved R23 and R109, and hydrophobic interactions between the naphthalene ring and the hydrophobic patch at the interface of the two helices forming the short arm of the L-shaped HMG boxes.

Moreover, PAM interacts with CXCL12 accommodating in the CXCR4 sulfoY21 binding site (Veldkamp et al. 2008), with its two salicylate moieties establishing polar interactions with R47, N44, and N45 side chains and with the backbone amide of Q48.

Importantly, in strong analogy to the other known HMGB1 ligands (including glycyrrhizin, Diflunisal and MCA), PAM inhibits HMGB1·CXCL12 heterocomplex formation and the heterocomplex-mediated chemotaxis (IC_50_ = 30 nM).

Indeed, the interaction of PAM with the HMGB1·CXCL12 heterocomplex does not exclude additional effects of PAM, as it often the case for salicylates (De Leo et al. 2019, 2020); in particular, PAM might affect the level or activity of other HMGB1 receptors, such as RAGE or TLR4. However, we deemed the activity of PAM on HMGB1-dependent chemotaxis of sufficient interest to warrant the exploration of its therapeutic potential in lung infections. We then used PAM both in the acute infection model established by direct intratracheal administration of the planktonic reference strain PAO1 and the chronic infection model established by the clinical MDR-RP73 strain embedded in agar beads (Bragonzi 2010; Cigana et al. 2020; Facchini et al. 2014; Kukavica-Ibrulj et al. 2014). Direct instillation by aerosol was preferred for local delivery of PAM as therapeutic agent into murine lung. Toxicity study showed no adverse effect for PAM up to 3 mM. In the acute infection model, PAM substantially reduced the inflammatory profile in the airways, particularly the neutrophil load, induced by *P. aeruginosa.* This is also strengthened by decreased MPO levels in different pulmonary districts including BALF and lung. This may reflect the contribution of immunomodulatory effects inhibiting HMGB1·CXCL12 dependent chemotaxis as suggested by in vitro data.

Previous studies in humans and murine models, including those from our group (Döring et al. 2014), have shown that therapeutic strategies that interfere with innate immune recruitment mechanisms have to be implemented with great caution since they harbor the risk of disabling innate host defense mechanisms and favoring risk of sepsis. Considering the potential interference of PAM with innate immune recruitment mechanisms, we evaluated the possible risk of favoring bacterial infections. In the acute infection model, PAM efficacy was associated with a modest increased bacterial burden, indicating a low risk of acute pulmonary exacerbation in a single-dose treatment. Next, we used a chronic infection model to evaluate whether PAM was still effective in a different dynamic model of cell mediated immunity and disease progression. We used the reported long-term chronic pulmonary infection murine model established previously to recapitulate the lung pathology of CF patients (Cigana et al. 2016). In the chronic model, mice treated by local (aerosol) repeated administration of PAM early after infection exhibited increased recovery and gain of body weight, compared to vehicle-treated animals, indicating improved health conditions. PAM was effective in reducing chronic airway murine inflammation induced by *P. aeruginosa* in a dose dependent manner, confirming results obtained in the acute infection model. In addition, PAM reduced also neutrophilic elastase suggesting limited tissue damage. Most importantly, the immunomodulator activity of PAM did not affect bacterial burden indicating that this regimen does not exacerbate the infection.

## Conclusions

In conclusion, our multidisciplinary approach encompassing structural studies, cellular assays, up to in vivo experiments, has provided evidence that PAM, a solubilizer commonly used in drug formulations, has translational potential as a small molecule with anti-inflammatory activity in the treatment of *P. aeruginosa* infection. This is of particular importance in chronic infection, as in patients with CF, where administration of compounds which interfere with the immune system may increase the risk of pulmonary exacerbation (Döring et al. 2014). PAM might be effective in CF patients with a mutation-agnostic/unknown profile, and might also cooperate with co-treatments with antibiotics and/or mucolytics and/or CFTR modulators. More studies are required, yet these results are good premises for the use of PAM in inflammatory pulmonary diseases, including COVID-19, where extracellular HMGB1 is expected to play a crucial role (Andersson et al. 2020).

## Supplementary Information

**Additional file 1.** Supplementary Methods (NMR measurements; Data Driven Docking Models and molecular images; Mouse Model; Bacteria preparation for acute infection; Agar beads preparation for chronic infection; Mice treatment with PAM), Supplementary Figures S1–S11 and Supplementary Tables S1–S2.

## Data Availability

The datasets used and/or analyzed during the current study are available from the corresponding author on reasonable request.

## Abbreviations

BALF: Bronchoalveolar lavage fluid
CF: Cystic fibrosis
CFUs: Colony-forming units
CFTCR: Cystic fibrosis transmembrane conductance regulator
COPD: Chronic obstructive pulmonary disease
CSP: Chemical shift perturbation
CXCL12: C-X-C Motif Chemokine Ligand 12
HMGB1: High Mobility Group B1
HSQC: Heteronuclear single quantum coherence
PAM: Pamoic acid
MPO: Myeloperoxidase
NMR: Nuclear Magnetic Resonance
NOE: Nuclear overhauser effect
RAGE: Receptor for advanced glycation endproducts
STD: Saturation Transfer Difference
TLR4: Toll like receptor 4
waterLOGSY: Water-Ligand Observed via Gradient SpectroscopY
