# Supplementary material for: Pamoic acid is an inhibitor of HMGB1·CXCL12 elicited chemotaxis and reduces inflammation in murine models of Pseudomonas aeruginosa pneumonia
Source: Mol Med. 2022 Sep 7;28:108. doi: 10.1186/s10020-022-00535-z (PMC9449960; doi:10.1186/s10020-022-00535-z)
Supplement: Supplementary file 1 — Additional file 1. Supplementary Methods (NMR measurements; Data Driven Docking Models and molecular images; Mouse Model; Bacteria preparation for acute infection; Agar beads preparation for chronic infection; Mice treatment with PAM), Supplementary Figures S1–S11 and Supplementary Tables S1–S2. [file 10020_2022_535_MOESM1_ESM.docx]

**Additional information**

**Pamoic acid is an inhibitor of HMGB1**•**CXCL12 elicited chemotaxis** **and reduces inflammation in murine models of *Pseudomonas aeruginosa* pneumonia**

Federica De Leo^1,2*^, Alice Rossi^3*^, Francesco De Marchis^2^, Cristina Cigana^3^, Medede Melessike^3^_,_ Giacomo Quilici^1^, Ida De Fino^3^_,_ Malisa Vittoria Mantonico^1,2^, Chantal Fabris^1^, Alessandra Bragonzi^3§^, Marco Emilio Bianchi^2§^, Giovanna Musco^1§^

^1^Biomolecular NMR Laboratory, Division of Genetics and Cell Biology, IRCCS San Raffaele Scientific Institute, Milano, Italy

^2^Università Vita-Salute San Raffaele, Milano

^3^Infection and Cystic Fibrosis Unit, Division of Immunology, Transplantation and Infectious Diseases, IRCCS San Raffaele Scientific Institute, Milano, Italy

^4^Chromatin Dynamics Unit, Division of Genetics and Cell Biology, IRCCS San Raffaele Scientific Institute, Milano, Italy

*Co-first authors

^§^To whom correspondence should be addressed: [bragonzi.alessandra@hsr.it](mailto:bragonzi.alessandra@hsr.it), [bianchi.marco@hsr.it](mailto:bianchi.marco@hsr.it), [musco.giovanna@hsr.it](mailto:musco.giovanna@hsr.it)

**ADDITIONAL MATERIAL AND METHODS**

**NMR measurements.** *Saturation Transfer Difference and Water-Ligand Observed via Gradient Spectroscopy*. STD and waterLOGSY experiments have been performed on 1 mM PAM in the presence of 0.05 mM HMGB1 in NMR buffer. STD experiments were acquired using a pulse scheme (Bruker pulse sequence: stddiffesgp.3) with excitation sculpting with gradients for water suppression and spin-lock field to suppress protein signals. The spectra were acquired using 128 scans, a spectral width of 9,600 Hz, and 64 K data points for acquisition. For protein saturation, a train of 60 Gaussian-shaped pulses of 50 ms was applied, for a total saturation time of 3 s. Relaxation delay was set to 3 s. On-resonance irradiations were set at 0 ppm. Off-resonance was always set at 107 ppm. STD spectra were obtained by internal subtraction of the on-resonance spectrum from the off-resonance spectrum. WaterLOGSY experiments were acquired using a pulse scheme as described (Dalvit et al. 2000) with excitation sculpting and flip-back for water suppression. The spectra were acquired using 128 scans, 32 K data points for acquisition, and mixing time was set to1s.

We also performed buildup STD experiments acquiring the STD spectra at seven different saturation times (t_sat_ = 0.5, 0.75, 1.25, 1.75, 2.5, 4, 6 s). For each resonance at each t_sat_ we calculated the STD amplification factor (STDamp) as

STD_amp_ = IntSTD / IntSTD_off_,

where IntSTD is the peak intensity in the STD spectrum and IntSTD_off_ is the intensity of the same peak in the off‐resonance spectrum. The STD_amp_ factors of each PAM proton were plotted against tsat and to fit the data of the buildup curve the following monoexponential equation was used:

STD_amp_ = STD_max_(1- e(-k_sat_))

where STD_max_ is the equilibrium STD intensity and k_sat_ is the rate constant of saturation transfer. Then the product between STD_max_ and k_sat_ represents the slope of each curve at t_sat_ = 0 as a T1-unbiased measure of the relaxation time. The slope values of each PAM proton were then normalized to percentage (STD %) to the maximum one (H4) (**Additional Table S1**).

*Titrations.* For NMR titrations, at each titration point a 2D water-flip-back ^1^H-^15^N-edited HSQC spectrum with 2,048 (160) complex points for ^1^H (^15^N), respectively, apodized by 90° shifted squared (sine) window functions, and zero filled to 256 points for indirect dimension. Assignment of the labeled proteins in the presence of the ligands (PAM or unlabeled protein) was obtained following individual cross-peaks through the titration series. For each residue, the weighted average of the ^1^H and ^15^N chemical shift perturbation (CSP) was calculated as CSP = [(Δδ^2^HN + Δδ^2^N/25)/ 2]^1/2^. PAM titrations have been performed on ^15^N HMGB1 full-length protein (20 mM phosphate buffer, pH 7.3, 150 mM NaCl, 1 mM DTT) and on ^15^N CXCL12 (20 mM phosphate buffer, pH 6, 20 mM NaCl) adding 0.25, 0.5, 0.75, 1, 2, 5 equivalents of PAM to the labeled proteins. To minimize dilution and NMR signal loss, titrations were carried out by adding small aliquots of concentrated PAM (10 mM in 20 mM phosphate buffer, pH 7.3, 150 mM NaCl) to the ^15^N-labeled protein samples (0.15 mM).

NMR-based antagonist-induced dissociation assays (Krajewski et al. 2007) were performed by stepwise additions of PAM (0.1, 0.2, 0.3, 0.4, 0.5, 0.75 and 1 equivalents) on ^15^N-HMGB1 in complex with CXCL12 (ratio 1:2) (20 mM phosphate buffer, pH 7, 150 mM NaCl, 308 K).

^1^H-^15^N-HSQC assignments of HMGB1 and CXCL12 were taken from the BMRB databank (HMGB1 accession numbers: 15148, 15149; CXCL12 accession number 16143) and confirmed via acquisition of 3D HNCA, CBCA(CO)NH experiments.

*^1^H resonance assignments of PAM* ^1^H resonance assignments of PAM (1 mM dissolved in NMR buffer) were obtained analyzing 2D TOCSY (Total correlation spectroscopy) (mixing time 50 ms) and NOESY (Nuclear Overhauser effect spectroscopy) (mixing time 400 ms) experiments.

Intermolecular nuclear Overhauser effect (nOes) between PAM and BoxA (residues 1-89) were obtained from 3D ^13^C-NOESY-HSQC with no evolution on ^13^C dimension (2,048 × 1 × 256 increments) experiments with ^15^N/^13^C filter in F1 (mixing time 200 ms); protein and ligand concentration were 0.8 and 1.6 mM, respectively, in D_2_O.

PAM protons were assigned by TOCSY experiments and 2D 1H-1H TOCSY (mixing time: 60 ms) and NOESY (mixing time: 120 ms) spectra.

*2D NMR lineshape analysis.* The NMR titrations of PAM on both ^15^N-HMGB1 and ^15^N-CXCL12 were in fast-intermediate exchange regime in the NMR time scale inducing significant line-shape broadening and peak intensity reduction, in particular at high stoichiometric ratio (1:2 and 1:5). We thus plotted the highest stoichiometric ratio at which we could still follow the CSPs (1:1) (**Figure 1A**). However, the line-broadening did not allow a proper K_d_ estimation from CSPs least-squares fitting. For this reason, we performed 2D NMR lineshape analysis using TITAN program (Waudby et al. 2016).

Selected residues with CSPs > Avg + SD of PAM-^15^N-HMGB1 (R9, Q20 and R23 in BoxA, S106, R109 and I158 in BoxB) and PAM-^15^N-CXCL12 (C11, K24, K27 and V49) interactions were fitted by optimizing the chemical shifts and line widths for the free and the bound state. The program estimates *K*_d_, *k*off and *n* parameters using a fixed stoichiometry of 1:2 and 1:1 binding models for HMGB1 and CXCL12, respectively. Error estimates for the fit-parameters were obtained using the bootstrap resampling of residuals procedure implemented in TITAN (Waudby et al. 2016).

**Data Driven Docking Models and molecular images.** Molecular docking of PAM on BoxA (residues G3-Y77) and BoxB (A93-G173), whose structures were extracted from 2YRQ (first structure of the NMR bundle), were performed using the data-driven software HADDOCK 2.2 (Dominguez et al. 2003; Van Zundert et al. 2016) and following the classical three-stage procedure, which includes: (1) randomization of orientations and rigid body minimization, (2) simulated annealing in torsion angle space, and (3) refinement in Cartesian space with explicit water. Ambiguous interaction restraints (AIRs) were defined as residues with CSP > Avg + SD were used to define active residues, whose solvent accessible surface neighbors were set as passive (**Additional Table S2**). In the case of CXCL12 (PDB 4UAI), only the residues located around the Diflunisal binding site (De Leo et al. 2019) were set as active (**Additional Table S2**). In the case of BoxA, intermolecular nOes were included as unambiguous restraints in the calculations only in the semi-flexible refinement stage, setting the maximum distance of the nOe H pairs to 5 Å (**Additional Table S2**).

Optimized parameters for liquid simulation (OPLS) were used for the protein (protein-allhdg5-4 and protein-allhdg5-4-caro). The geometric coordinates and parameters for PAM were calculated and optimized using the PRODRG server (Schüttelkopf and Van Aalten 2004). Calculations generated 1,000, 1,000, and 500 structures for the rigid body docking (it0), the semi-flexible refinement (it1), and the explicit solvent refinement (water), respectively. The final 500 structures obtained after water refinement were scored with HADDOCK (HADDOCKscore = 1.0 EvdW + 0.2 Eelec + 1.0 Edesolv + 0.1 EAIR) for a weighted combination of van der Waals (vdW) and electrostatic energy terms (Lennard–Jones and Coulomb potentials), empirical desolvation term (Fernández-Recio et al. 2004), and ambiguous interaction restraint energy term, which reflects the accordance of the model to the input restraints.

HADDOCK models were clustered based on their interface root mean square deviation (rmsd), setting the cutoff and the minimum number of models in a cluster to 2 Å and 5 for the boxes, and 2 Å and 3 for CXCL12, respectively. Proteins were aligned and fitted on the backbone of active residues, reported in **Additional Table S2**. The rmsd of PAM was calculated only on the heavy atoms of the entire scaffold.

To remove any bias of the cluster size on the cluster statistics, the final overall score of each cluster was calculated on the four lowest HADDOCK scores models in that cluster. For each protein the cluster with the best fitting relative to the experimentally-driven restraints (lowest number of violations) and the best HADDOCK score (cluster 1 for BoxA and CXCL12, cluster 2 for BoxB) was selected (**Additional Figure S3-4 and 7**).

The analysis of the docking calculations was performed applying in-house python and tcl scripts.

**Mouse model.** Immunocompetent C57BL/6NCrlBR male mice (8-10 weeks of age) were purchased from Charles River (Calco, Italy), shipped in protective, filtered containers, transported in climate-controlled trucks, and allowed to acclimatize for at least two days in the stabulary prior to use. Mice were maintained in the biosafety level 3 (BSL3) facility at San Raffaele Scientific Institute (Milano, Italia) where 3-5 mice per cage were housed. Mice were maintained in sterile ventilated cages. Mice were fed with standard rodent autoclaved chow (VRFI, Special Diets Services, UK) and autoclaved tap water. Fluorescent lights were cycled 12h on, 12h off, and ambient temperature (23 ± 1°C) and relative humidity (40-60%) were regulated.

For infection experiments, mice were anesthetized by an intraperitoneal injection of a solution of Avertin (2,2,2- tribromethanol, 97%) in 0.9% NaCl and administered at a volume of 0.015 ml/g body weight. Mice were placed in supine position. The trachea was directly visualized by ventral midline, exposed and intubated with a sterile, flexible 22-g cannula attached to a 1 ml syringe. An inoculum of 60 μl of planktonic bacterial cells or 50 μl of agar bead suspension was implanted via the cannula into the lung. After inoculation, all incisions were closed by suture.

Mice were monitored daily for coat quality, posture, attitude, ambulation, hydration status and body weight. Mice that lost >20% body weight and had evidence of severe clinical disease, such as scruffy coat, inactivity, loss of appetite, poor locomotion, or painful posture, were sacrificed before the termination of the experiments with an overdose of carbon dioxide. Gross lung pathology was noted.

Broncho alveolar lavage fluid (BALF) was extracted with a 22-gauge venous catheter, ligated to the trachea to prevent backflow. The lungs were washed with three one ml of RPMI-1640 (Euroclone) with protease inhibitors (Complete tablets, Roche Diagnostic) and pooled. Quantitative bacteriology on BALF was performed by plating serial dilution on tryptic soy agar (TSA). Total cells present in the BALF were counted using an inverted light optical microscope after diluting an aliquot of the BALF 1:2 with Tuerk solution in a disposable counting chamber. BALF cells were centrifuged at 330 x g for 8 min at 4°C. If the pellet was red, erythrocytes were lysed by resuspending the pellet in 250- 300 µl of RBC lysis buffer diluted 1:10 in ultra-pure distilled water for 3 min. Then, 2-3 ml PBS were added and cells were centrifuged at 330 x g for 8 min at 4°C. The pellet was resuspended in RPMI 1640 10% fetal bovine serum (FBS) at concentration of 1x10^6^ cells/ml, and an aliquot of 150 µl was pipetted into the appropriate wells of the cytospin and centrifuged at 300 x g for 5 min medium brake. Slides were then stained by Diff-Quik staining using a commercial kit (Medion Diagnostics, code: 726443), according to the manufacturer’s instructions. A differential cell count was performed at an inverted light optical microscope.

Lungs were excised aseptically and homogenized in 2 ml PBS added with protease inhibitors using the homogenizer gentleMACS^TM^ Octo Dissociator. One-hundred μl of the homogenates and 10-fold serial dilutions were spotted onto TSA. CFU were determined after overnight growth at 37°C.

Infections, treatments and sacrifices in the chronic infection models were all performed in the late morning, while the sacrifices in the acute infection model were performed in the late afternoon. In addition, in all the experiments, mice had been subdivided according to the body weight to have similar mean in all the groups of treatment.

Animal studies were conducted according to protocols approved by San Raffaele Scientific Institute (Milan, Italy) Institutional Animal Care and Use Committee (IACUC #733) and adhered strictly to the Italian Ministry of Health guidelines for the use and care of experimental animals.

**Bacteria preparation for acute infection.** An aliquot of *P. aeruginosa* PAO1 reference strain from glycerol stocks was streaked for isolation on tryptic soy agar (TSA) and incubated at 37°C O/N. Bacterial glycerol stocks were not used more than three times to avoid variability in the animal experiments. One colony was picked from the plate and used to inoculate 5 ml of tryptic soy broth (TSB) (BD, Becton and Dickinson) and placed in a shaking incubator at 37°C 200 rpm O/N. The O/N bacterial suspension was diluted to 0.1 OD/ml in 20 ml of TSB / flask and grown for 3 h at 37°C at 200rpm, to reach the log phase (Cigana et al. 2016; Lorè et al. 2012). The bacteria were pelleted by centrifugation (2,700 *g*, 15 min, 4°C), resuspended in sterile phosphate-buffered saline (PBS) and diluted to give the required dose in 60 µl (1x10^6^ colony forming units - CFU). Mice were anaesthetized by intraperitoneal injection as described before, then infected intratracheally with *P. aeruginosa* (planktonic form).

**Agar beads preparation for chronic infection.** The agar beads *P. aeruginosa* mouse model was used (Facchini et al. 2014) (Cigana et al. 2016)(Bragonzi et al. 2009). An aliquot of *P. aeruginosa* MDR-RP73 clinical strain from glycerol stocks was streaked for isolation on TSA and incubated at 37°C O/N. One colony was picked from the plate and used to inoculate 10 ml of TSB and placed in a shaking incubator at 37°C 200 rpm O/N. The O/N bacterial suspension was diluted to 0.15 OD/ml in 20 ml of TSB / flask and grown for 4 h at 37°C at 200rpm, to reach the log phase. The bacteria were pelleted by centrifugation (2,700 *g*, 15 min, 4°C) and resuspended in 1 ml PBS (pH 7.4). A starting amount of 2 x10^9^ CFU of *P. aeruginosa* was used for inclusion in the agar beads prepared according to the previously described method (Cigana et al. 2016; Facchini et al. 2014). Bacteria were added to 9 ml of 1.5% TSA (w/v), prewarmed to 50°C. This mixture was pipetted forcefully into 150 ml heavy mineral oil at 50°C and stirred rapidly with a magnetic stirring bar for 6 min at room temperature, followed by cooling at 4°C with continuous slowly stirring for 20 min. The oil-agar mixture was centrifuged at 2700 g for 15 min to sediment the beads, and washed six times in PBS. The size of the beads was verified microscopically and only those preparations containing beads of 100 μm to 200 μm in diameter were used as inoculum for animal experiments. The number of *P. aeruginosa* CFU in the beads was determined by plating serial dilutions of the homogenized bacteria-bead suspension on TSA plates. The inoculum was prepared by diluting the beads suspension with PBS to 1x10^7^ CFU/ml, to inoculate about 5x10^5^ CFU/50μl. *P. aeruginosa* beads were prepared the day before inoculation, stored overnight at 4°C for a maximum of two days. The number of *P. aeruginosa* CFU in the beads inoculated was determined by plating serial dilutions of the homogenized bacteria-bead suspension on TSA plates of the aliquot used for infection experiment at the day of the infection.

**Mice treatment with PAM.** PAM was diluted in PBS for injection. For toxicity and efficacy experiments, animals were separated into three groups (two doses of PAM, and vehicle). PAM therapy was initiated five min after the i.t. surgery and was repeated once a day for seven days for chronic infection. Mice were treated with PAM or vehicle (PBS) by local administration using Penn-Century **MicroSprayer® Aerosolizer** (p.c., volume: 50 μl). Endotracheal administration with p.c. was carried out under 5% isoflurane–oxygen. PAM toxicity during treatment was evaluated by a general mouse observation in the cage with cage mates. Next, the mouse was transferred to a new cage and mouse observation was started. A unique score was assigned based on clinical symptoms including ruffled hair, kyphosis, abnormal behavior (e.g. less movement, less exploratory and interactive with the new environment), dyspnea and torpor. In addition, body weight and rectal body temperature using thermometer were measured during the follow up.

For efficacy studies, mice were humanely euthanized at different time points, to determine efficacy of treatment, in accordance with animal welfare regulations.

**ADDITIONAL BIBLIOGRAPHY**

Bragonzi A, Paroni M, Nonis A, Cramer N, Montanari S, Rejman J, et al. Pseudomonas aeruginosa Microevolution during Cystic Fibrosis Lung Infection Establishes Clones with Adapted Virulence. Am. J. Respir. Crit. Care Med. 2009 Jul 15;180(2):138–45.

Cigana C, Lorè NI, Riva C, De Fino I, Spagnuolo L, Sipione B, et al. Tracking the immunopathological response to Pseudomonas aeruginosa during respiratory infections. Sci. Rep. 2016 Aug 17;6(1):21465.

Dalvit C, Pevarello P, Tato M, Veronesi M, Vulpetti A, Sundström M. Identification of compounds with binding affinity to proteins via magnetization transfer from bulk water. J. Biomol. NMR. 2000;18(1):65–8.

Dominguez C, Boelens R, Bonvin AMJJ. HADDOCK: A protein-protein docking approach based on biochemical or biophysical information. J. Am. Chem. Soc. 2003;125(7):1731–7.

Facchini M, De Fino I, Riva C, Bragonzi A. Long Term Chronic Pseudomonas aeruginosa Airway Infection in Mice. J. Vis. Exp. 2014 Mar 17;(85).

Fernández-Recio J, Totrov M, Abagyan R. Identification of Protein–Protein Interaction Sites from Docking Energy Landscapes. J. Mol. Biol. 2004 Jan 16;335(3):843–65.

Krajewski M, Rothweiler U, D’Silva L, Majumdar S, Klein C, Holak TA. An NMR-based antagonist induced dissociation assay for targeting the ligand-protein and protein-protein interactions in competition binding experiments. J. Med. Chem. 2007;50(18):4382–7.

De Leo F, Quilici G, Tirone M, De Marchis F, Mannella V, Zucchelli C, et al. Diflunisal targets the HMGB1/CXCL12 heterocomplex and blocks immune cell recruitment. EMBO Rep. 2019 Oct 4;20(10):e47788.

Lorè NI, Cigana C, De Fino I, Riva C, Juhas M, Schwager S, et al. Cystic Fibrosis-Niche Adaptation of Pseudomonas aeruginosa Reduces Virulence in Multiple Infection Hosts. Cornelis P, editor. PLoS One. 2012 Apr;7(4):e35648.

Schüttelkopf AW, Van Aalten DMF. PRODRG: A tool for high-throughput crystallography of protein-ligand complexes. Acta Crystallogr. Sect. D Biol. Crystallogr. 2004 Aug 1;60(8):1355–63.

Waudby CA, Ramos A, Cabrita LD, Christodoulou J. Two-Dimensional NMR Lineshape Analysis. Sci. Rep. 2016 Jul 25;6(1):24826.

Van Zundert GCP, Rodrigues JPGLM, Trellet M, Schmitz C, Kastritis PL, Karaca E, et al. The HADDOCK2.2 Web Server: User-Friendly Integrative Modeling of Biomolecular Complexes. J. Mol. Biol. Academic Press; 2016 Feb 22;428(4):720–5.

**Additional Figures**

**
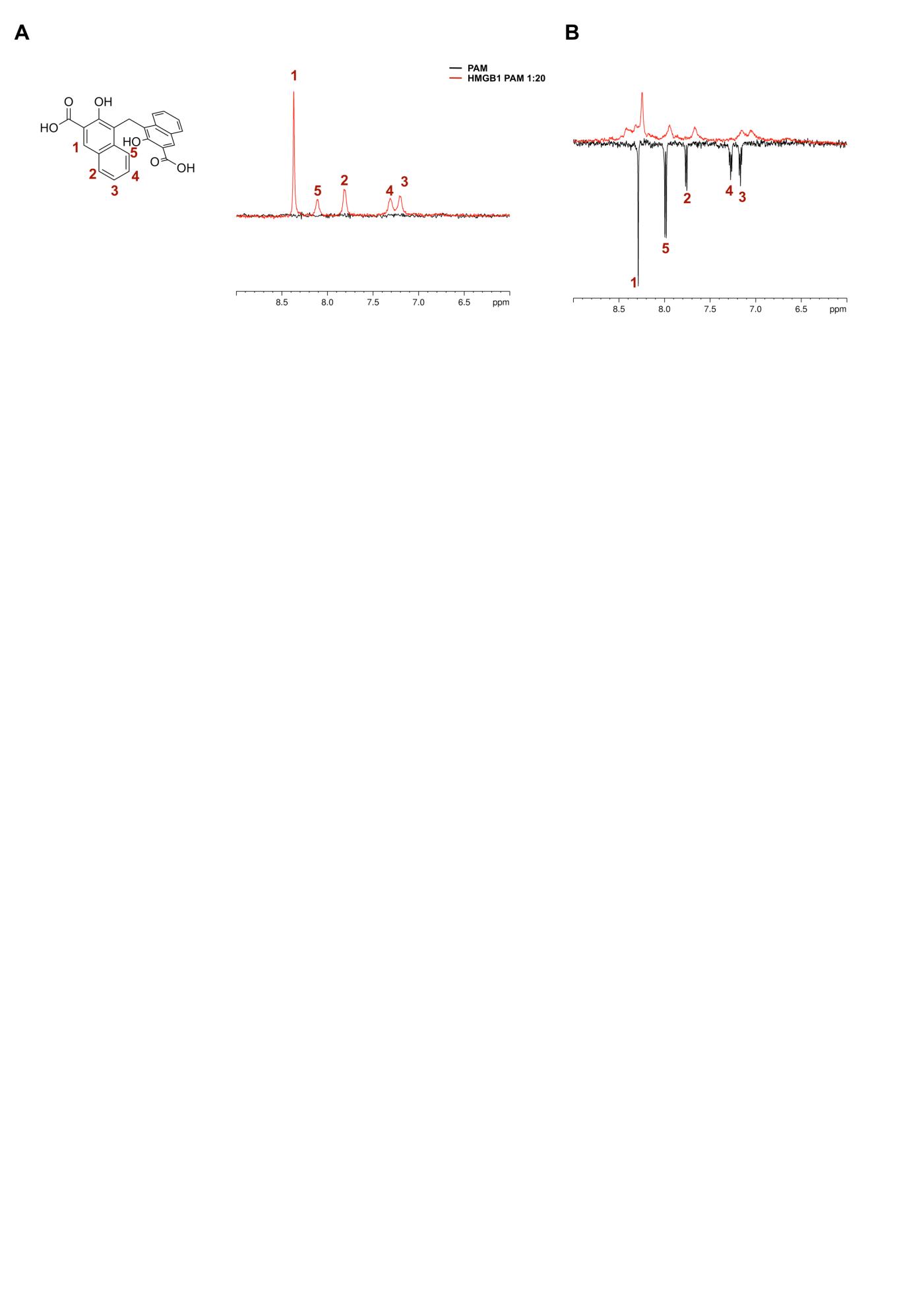
**

**Figure S1**. **STD and WaterLOGSY experiments performed on PAM in the presence of HMGB1.** A) Saturation Transfer Difference (STD) results obtained for 1 mM PAM alone (black line) and with 0.05 mM HMGB1 (red line) in 20 mM phosphate buffer, 150 mM NaCl, 1 mM DTT pH 7.3 (on resonance: 0 ppm; saturation time: 3 s). STD signals with different intensities are observed for all protons in the presence of HMGB1, indicating that PAM directly binds to HMGB1. The numbered peaks correspond to proton resonance assignments on PAM chemical structure. B) WaterLOGSY spectra obtained for 1 mM PAM alone (black line) and in complex with 0.05 mM HMGB1 (red line). All PAM protons display signals inversion, indicating binding to HMGB1.

**
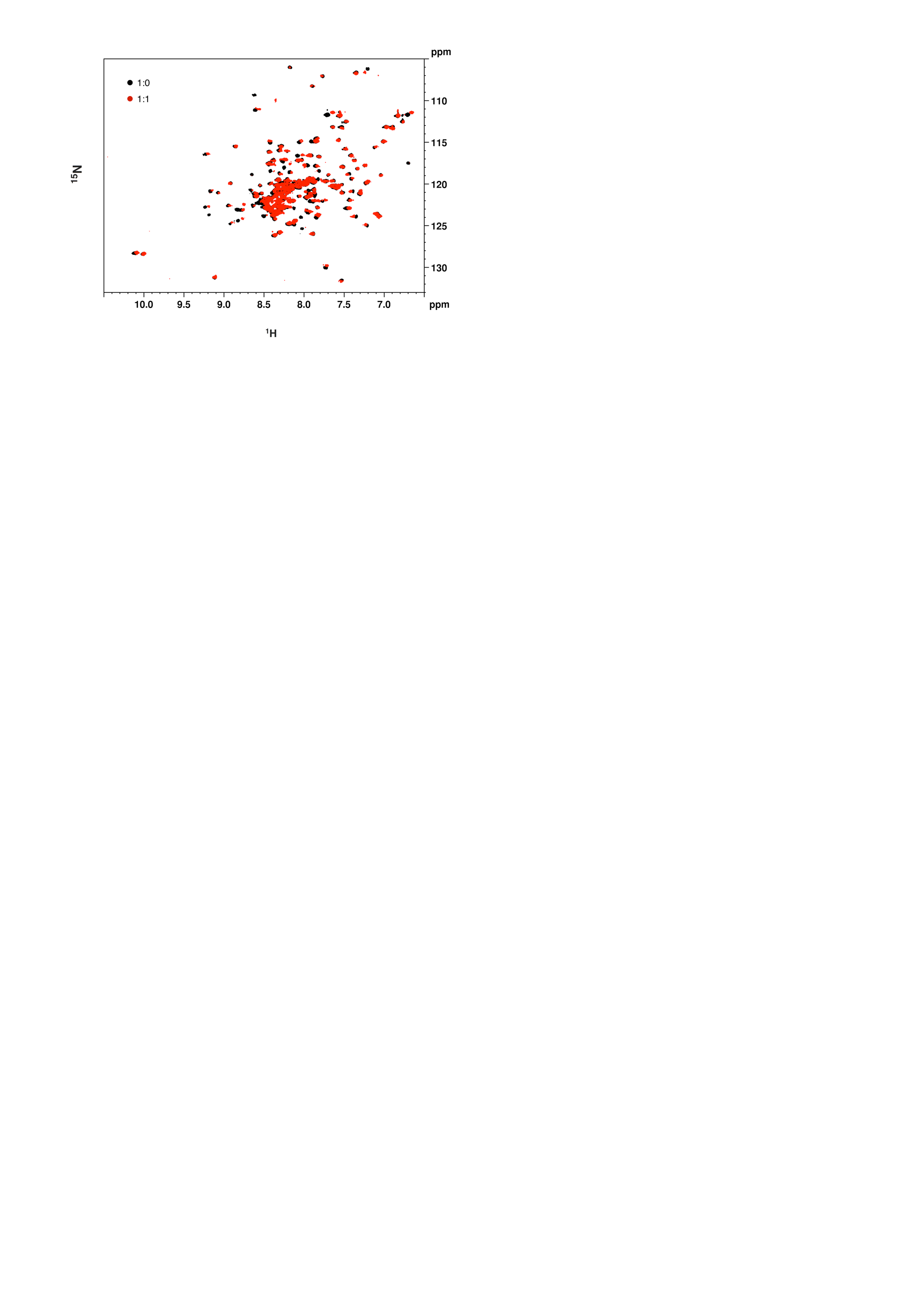
**

**Figure S2. 2D ^1^H-^15^N HSQC spectra of HMGB1 without and with PAM.** Superposition of the ^1^H-^15^N HSQC spectra of HMGB1 (0.15 mM) without (black) and with (red) equimolar concentration of PAM, 20 mM phosphate buffer, pH 7.3, 150 mM NaCl, 1 mM DTT.

**
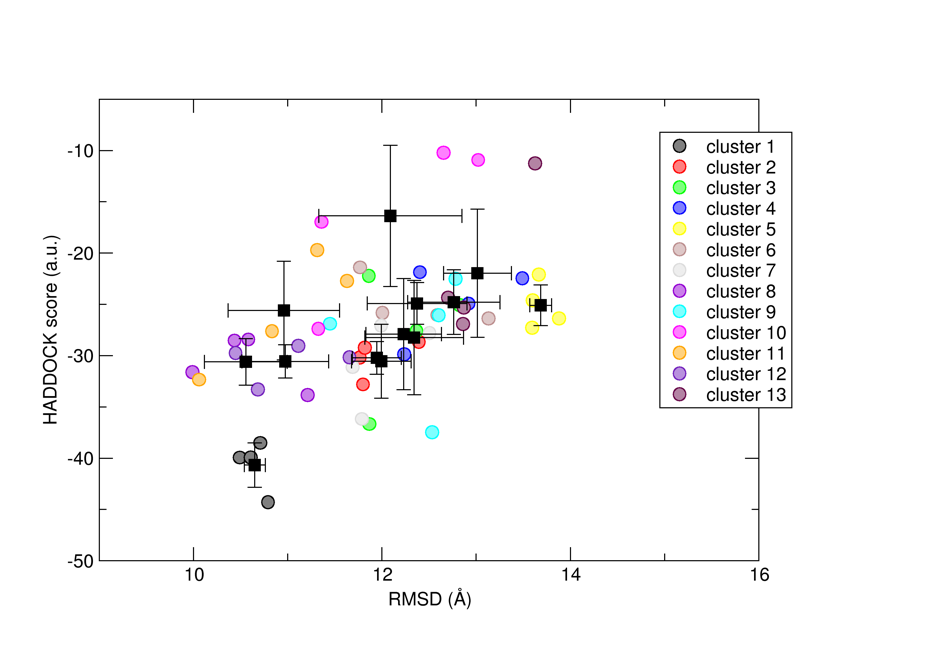
**

**Figure S3.** **HADDOCK score plot of BoxA and PAM.** HADDOCK score *versus* rmsd from the lowest Haddock energy complex structure between BoxA and PAM. Circles correspond to the four best structures in each cluster, the cluster averages with the standard deviation are indicated with the black squares and bars.

**
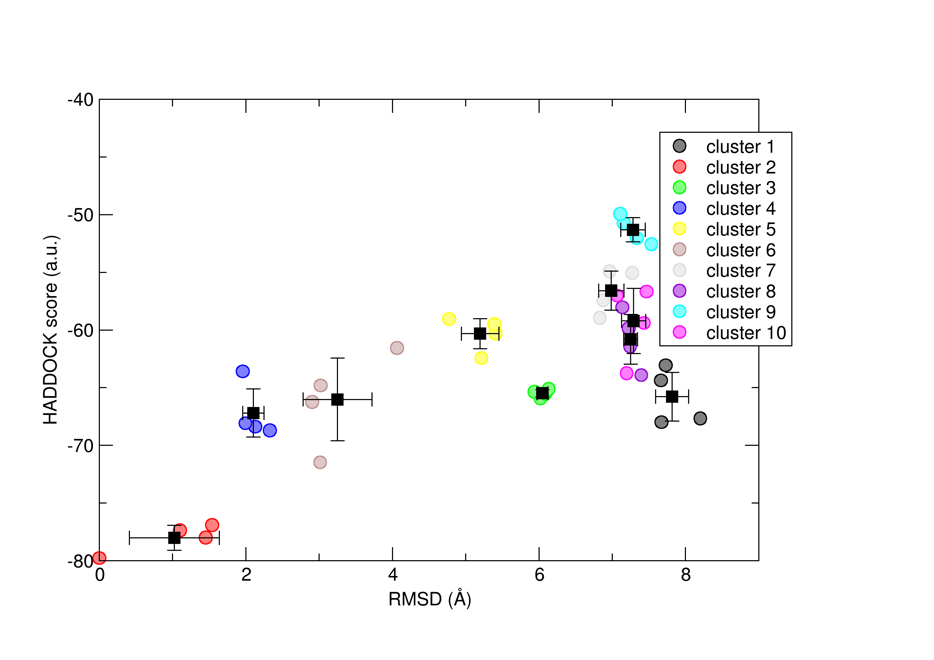
**

**Figure S4.** **HADDOCK score plot of BoxB in complex with PAM.** HADDOCK score *versus* rmsd from the lowest Haddock energy complex structure between BoxB and PAM. Circles correspond to the four best structures in each cluster, the cluster averages with the standard deviation are indicated with the black squares and bars.

**
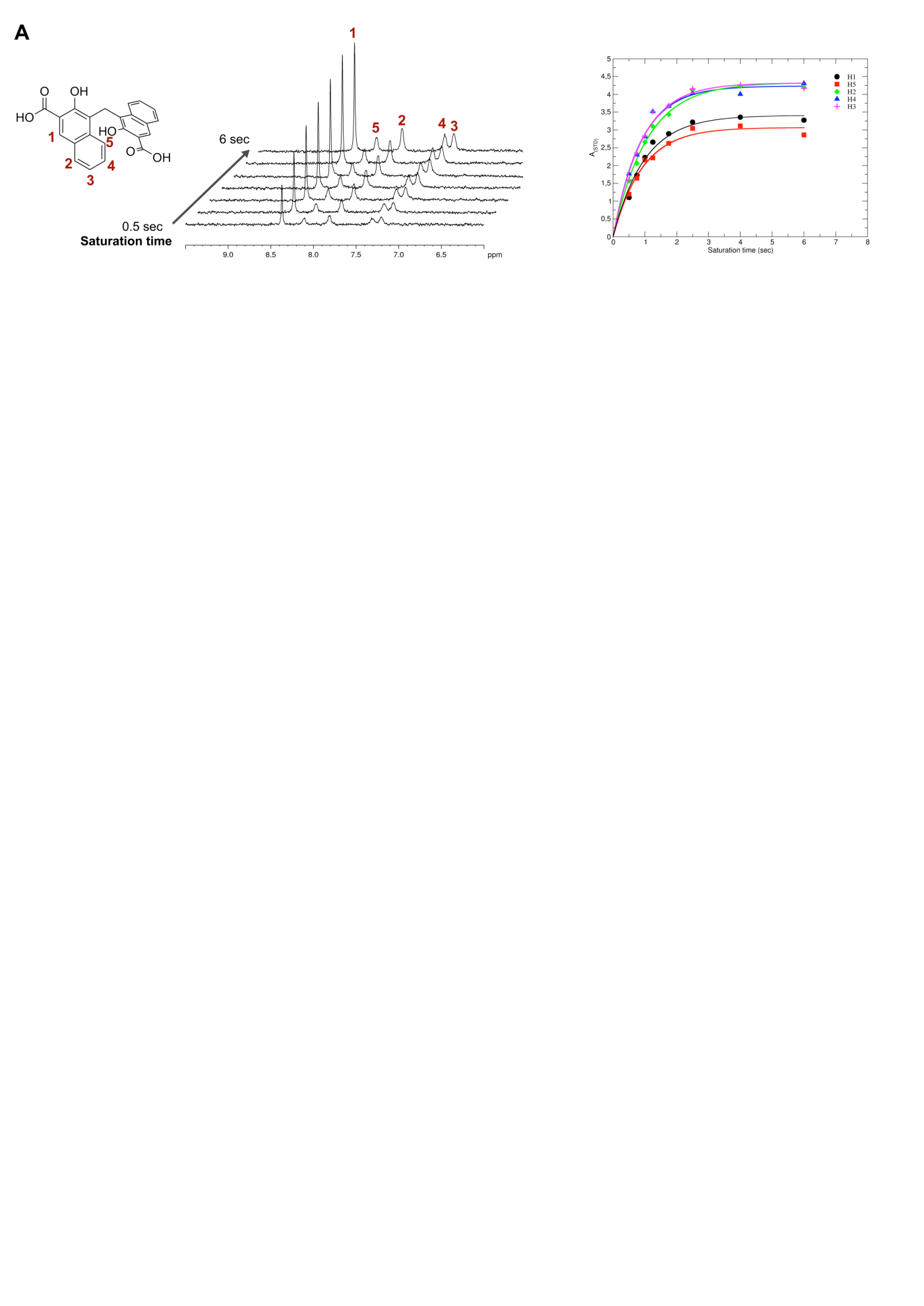
**

**Figure S5**. **STD buildup experiments** A) Stacked 1D STD spectra obtained for 1 mM PAM with 0.05 mM HMGB1 (red line) at increasing saturation time (from bottom to top: 0.5, 0.75, 1.25, 1.75, 2.5, 4, 6 s) in 20 mM phosphate buffer, 150 mM NaCl, 1 mM DTT pH 7.3 (on resonance: 0 ppm). The numbered peaks correspond to proton resonance assignments on PAM chemical structure. Right: STD build-up curves for five PAM protons with as a function of the saturation time. Experimental data were fit to a rising exponential to obtain STD_max_ and the saturation rate constant k_sat_.


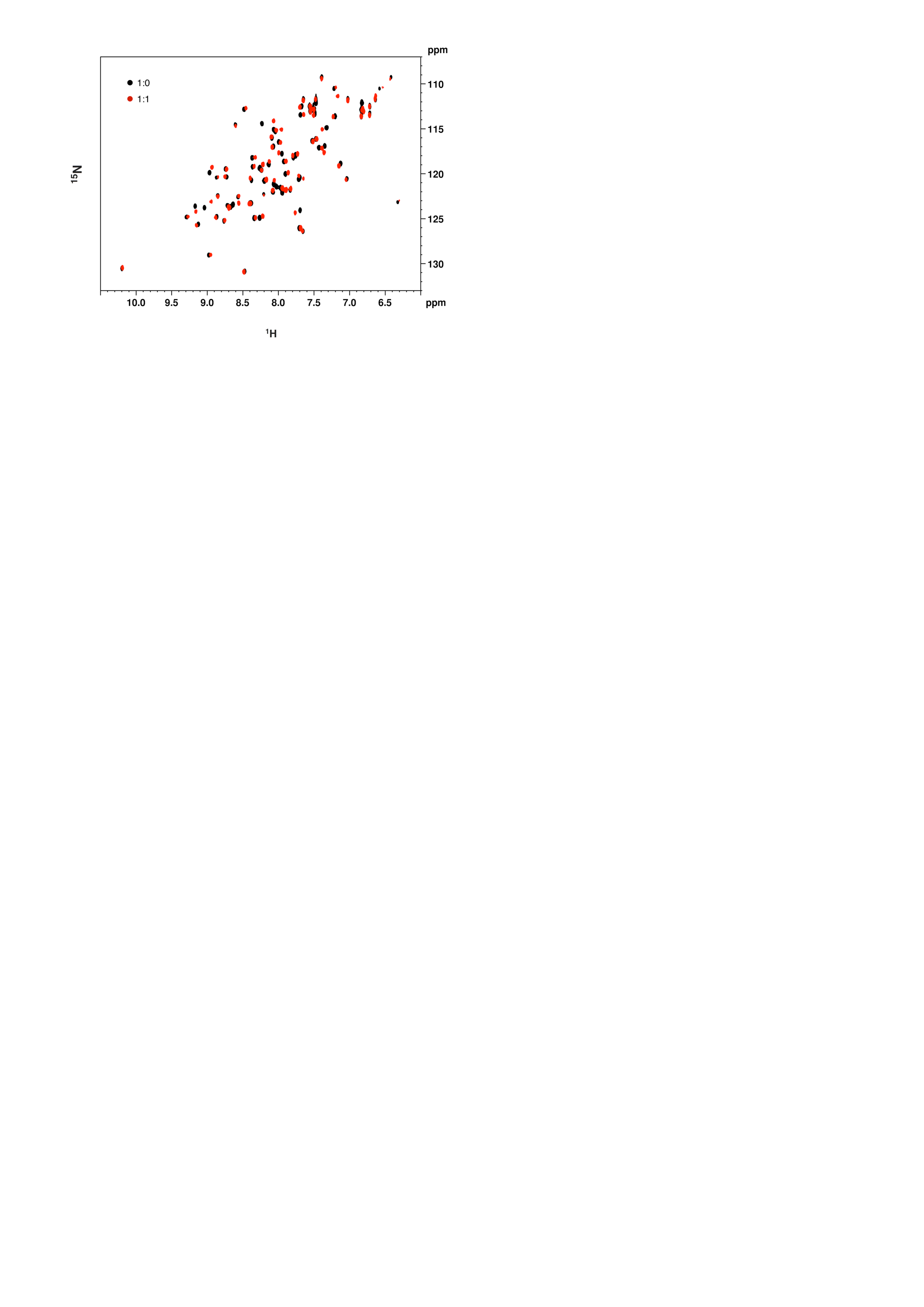


**Figure S6**: **2D ^1^H-^15^N HSQC spectra of CXCL12 without and with PAM.** Superposition of the ^1^H-^15^N HSQC spectra of CXCL12 (0.15 mM) without (black) and with (red) equimolar concentration of PAM, 20 mM phosphate buffer, pH 6.5, 20 mM NaCl

**
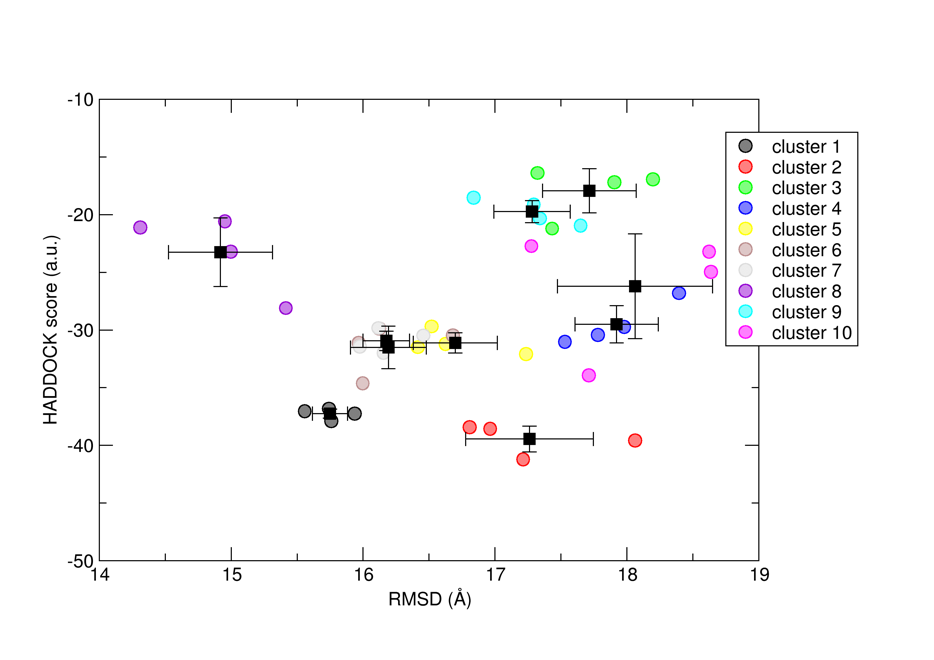
**

**Figure S7.** **HADDOCK score plot of CXCL12 in complex with PAM** HADDOCK score *versus* rmsd from the lowest HADDOCK energy complex structure between CXCL12 and PAM. Circles correspond to the four best structures in each cluster, the cluster averages with the standard deviation are indicated with the black squares and bars.

**
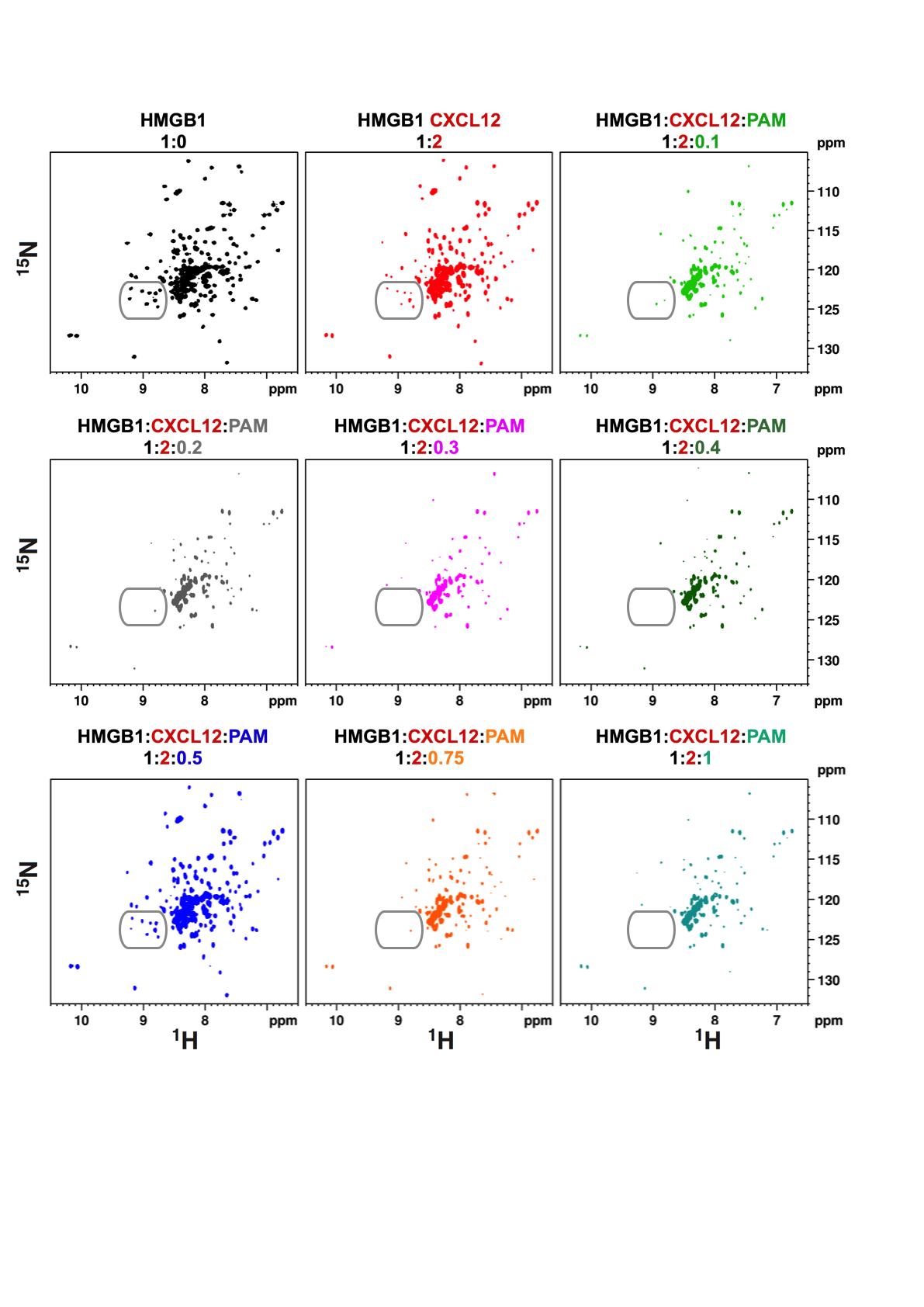
Figure S8**: **Effect of PAM on 2D ^1^H-^15^N HSQC spectra of HMGB1**•**CXCL12.** ^1^H-^15^N HSQC HMGB1 (0.1 mM) spectrum (black) and with 0.2 mM of CXCL12 (red). On the preformed HMGB1•CXCL12 complex increasing stochiometric ratio of PAM was stepwise added 0.1 (green spectrum), 0.2 (gray), 0.3 (magenta), 0.4 (dark green), 0.5 (blu), 0.75 (orange) and 1 (teal), (20 mM phosphate buffer, pH 7, 150 mM NaCl, 308 K). At 0.5 stoichiometric ratio a significant recovery of the HMGB1 resonances is observed. At higher stoichiometric ratio line broadening effects occur, due to the interaction of PAM with HMGB1, which occurs in the fast-intermediate exchange regime in the NMR time scale. The rectangles highlight the region reported in Figure 1C.

**
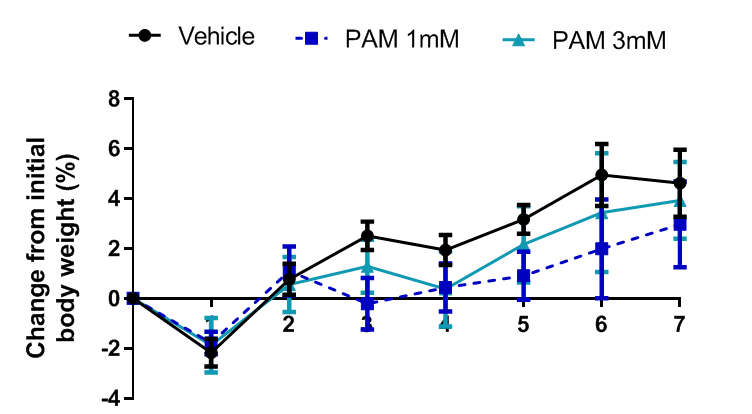
**

**Figure S9. Toxicity of PAM after repeated aerosol treatment in mice.** C57BL/6NCrlBR male mice (aged 8–10 weeks) received intratracheal inoculation with PBS to mimic the surgery applied to infected mice. Treatment started five minutes after surgery, with PAM (1 mM and 3 mM) or vehicle administered *via* aerosol by Penn Century daily for seven days. Before each administration, mice were weighted, and the percentage change from the initial body weight was averaged for each group of mice. Data are presented as mean ± SEM. Data are pooled from 4 mice/group.


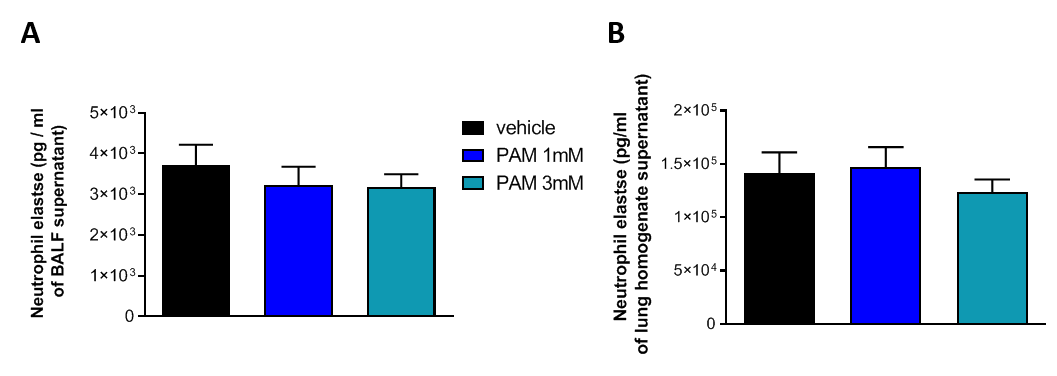
**Figure S10. Efficacy of aerosol treatment with PAM (1 mM and 3 mM) in a mouse model of acute *P. aeruginosa* airway infection**. C57BL/6NCrlBR male mice (aged 8–10 weeks) received intratracheal inoculation with 1×10^6^ CFU of planktonic PAO1 strain. Five minutes after infection, PAM 1 mM, PAM 3 mM or vehicle were administered *via* an aerosolizer. After six hours, mice were sacrificed, bronchoalveolar lavage fluid (BALF) was collected, and the lungs were excised and homogenized. Neutrophils elastase concentration was evaluated in the supernatants of BALF (**A**) and lung homogenate (**B**) by ELISA assay. Data are presented as mean ± SEM. Data are pooled from three independent experiments (n=14-15 mice).


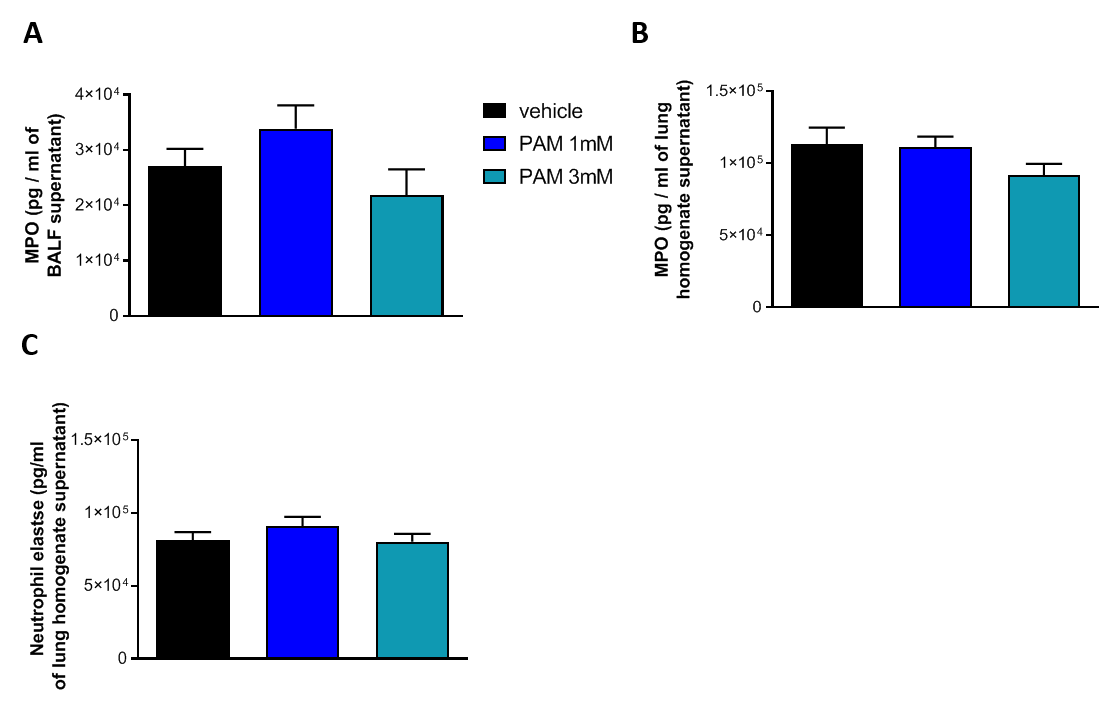


**Figure S11. Efficacy of aerosol treatment with PAM (1 mM and 3 mM) in a murine model of *P. aeruginosa* MDR-RP73 chronic airways infection**. C57BL /6NCrlBR male mice (aged 8–10 weeks) received intratracheal inoculation with 5×10^5^ CFU of MDR-RP73 strain embedded in agar beads. Treatment started five minutes after infection, with PAM (1 mM and 3 mM) or vehicle administered *via* an aerosolizer daily for seven days. At day seven post-infection, mice were sacrificed, BALF was collected and lungs were excised and homogenized. MPO concentration was evaluated in the supernatant of BALF (**A**) and lung homogenate (**B**) and neutrophils elastase concentration was evaluated in the supernatants of lung homogenate (**C**) by ELISA assay. Data are presented as mean ± SEM. Data are pooled from three independent experiments (n=15-16 mice).

**Additional** **Table S1**. The slope values of each PAM proton as determined by fitting the STD build-up curves. The slope values are normalized to percentage (STD %) to the maximum proton (H4). The symbols are the same as in **Figure S2**.

| **Proton** | **Slope** | **%** |
| --- | --- | --- |
| **H1** | 3.5 | 73 |
| **H2** | 4.1 | 85 |
| **H3** | 4.7 | 98 |
| **H4** | 4.8 | 100 |
| **H5** | 3.3 | 69 |

**Additional** **Table S2**. List of Ambiguous Interaction Restraints (AIRs) and of Unambiguous (nOe) restraints used in HADDOCK calculations.

|  | Ambiguous | | Unambiguous^a,b^ |
| --- | --- | --- | --- |
| Domain | Active | Passive |  |
| Box A | Y15, A16, F17, F18, V19^nOe^, Q20, R23, V35 ^nOe^, F37 ^nOe^, S41, S45 | R9, Y15, F17, F18, V19, Q20, R23, E24, H26, K27, V35, F37, E40, S41, C44, W48 | HG*_V19_-H3 _PAM_;  or HG*_V19_-H4 _PAM_;  or HD*_F37_-H3/4_PAM_  or HE*_F37_-H3/4_PAM_;  HGA_V35_-H3/4 _PAM_ |
| Box B | C105, R109, I112, K113, I121, V124, A125, K127, L128 | F102, F104, S106, H116, G118, L119, S120, D123, K126, G129, E130 | *n.d.* |
| CXCL12 | E15, 17, V18, N45, R47, Q48, V49 | S4, 7, 9, C11, V23, K24, H25, L26, K27, Q48 | *n.d.* |
| *n.d.* Not determined  ^a^ OPLS *force field* nomenclature of protein hydrogens  ^b^ PAM hydrogens (Figure S1) | | | |
